# The complete mitochondrial genome of the *Boulenophrys xianjuensis* (Anura: Megophryidae)

**DOI:** 10.1080/23802359.2026.2694182

**Published:** 2026-06-25

**Authors:** Yingying Xing, Huimin Pei, Haili Ou, Lei Xie, YongPu Zhang

**Affiliations:** a College of Life and Environmental Science, Whenzhou University, Wenzhou, China; b Zhejiang Provincial Key Laboratory for Subtropical Water Environment and Marine Biological Resources Protection, Wenzhou University, Wenzhou, China

**Keywords:** Mitogenome, phylogenetic analysis, next-generation sequencing

## Abstract

*Boulenophrys xianjuensis* is a member of the family Megophryidae and was first described in 2020 in Xianju County, Zhejiang Province, China. In this study, the complete mitochondrial genome of *B. xianjuensis* was sequenced, assembled, and annotated using next-generation sequencing technology. The assembled mitogenome is a circular molecule 17,602 base pairs (bp) in length, with a GC content of 41.40%, and contains 13 protein-coding genes (PCGs), two rRNA genes, 22 tRNA genes, and a single control region (D-loop). The majority of PCGs are encoded on the heavy strand (H-strand), whereas one PCG (*ND6*) and eight tRNA genes (*trnE*(uuc), *trnS*(tga), *trnY*(gua), *trnC*(gca), *trnN*(guu), *trnA*(ugc), *trnQ*(uug), and *trnP*(ugg)) are encoded on the light strand (L-strand). Phylogenetic analysis placed *B. xianjuensis* within a well-supported clade comprising other *Boulenophrys* species, with the genus *Boulenophrys* forming a sister group to *Atympanophrys*. This new newly characterized mitogenome provides valuable molecular data for understanding the mitochondrial genome of *B. xianjuensis* and contributes significantly to the clarification of the phylogenetic relationships within the genus *Boulenophrys*.

## 1. Introduction

The genus *Boulenophrys*, comprising 77 recognized species worldwide, belongs to the family Megophryidae, order Anura, and class Amphibia (Frost 2025). These species are predominantly distributed across various regions of China, with the ranges of some species extending southward into Vietnam, Laos, Thailand, and Myanmar (Frost 2025). In 2020, the description of the newly identified species *Boulenophrys xianjuensis* (Wang, Wu, Peng, Shi, Lu, and Wu 2020)—endemic to Xianju County, Zhejiang Province, China—contributed to the growing diversity of the genus (Wang et al. 2020). However, knowledge of *B. xianjuensis* remains limited due to the paucity of genetic data, which currently consists only of partial mitochondrial DNA sequences of the *rrn16* and *cox1* genes (Wang et al. 2020), while the complete mitochondrial genome has not yet been characterized. Additionally, *B. xianjuensis* is currently known only from its type locality and has not yet been assessed by the IUCN Red List. To date, the GenBank database includes complete mitochondrial genomes for only seven other *Boulenophrys* species: *Boulenophrys kuatunensis* (Pope 1929), *Boulenophrys baishanzuensis*, *Boulenophrys boettgeri*, *Boulenophrys sangzhiensis*, *Boulenophrys spinata*, *Boulenophrys tuberogranulata*, and *Boulenophrys omeimontis*. To bridge this gap in mitochondrial genome, we successfully sequenced and analyzed the complete mitochondrial genome (mitogenome) of *B. xianjuensis* using next-generation sequencing technology. Comparative sequence analysis was conducted with other species within the family Megophryidae to elucidate the phylogenetic relationships.

## 2. Materials and methods

### 2.1. Sample collection

**Figure 1.**
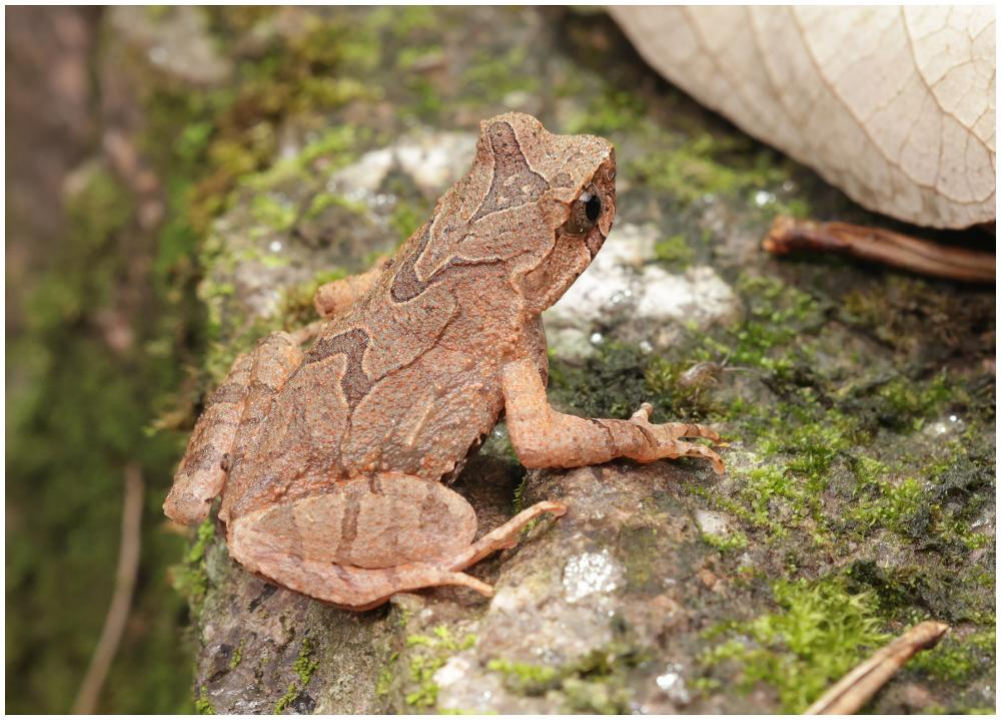
Male adult specimen of *Boulenophrys xianjuensis* used in this study, photographed by Yongpu Zhang.

In September 2024, a male adult specimen of *B. xianjuensis* specimen (Figure 1) was collected from Qinglonggu, Yongjia County, Zhejiang Province, China (28.55°N, 120.80°E; elevation 875 m). The specimen was morphologically identified, and approximately 20 mg of muscle tissue was subsequently excised for DNA extraction. To confirm species identity, we provided molecular evidence. Based on comparisons of partial sequences of *cox1* gene, the sequence similarities between the object specimen and three reference sequences of *B. xianjuensis* from GenBank ranged from 99.10% to 99.82%. Morphologically, the specimen also matched the diagnostic characteristics of this species: (1) vomerine ridge present, vomerine teeth absent; (2) posterior edge of tongue not notched; (3) a small horn-like tubercle on upper eyelid; (4) tympanum distinct, rounded; (5) two metacarpal tubercles; (6) finger length order II < I < IV < III; (7) rudimentary webbing at toe bases; (8) heels overlapping when hind limbs flexed at right angles; (9) tibio-tarsal articulation reaching between tympanum and eye; (10) male with a single internal subgular vocal sac; (11) male with nuptial pads and spines on first and second fingers during breeding season. Consequently, we can conclusively identify the specimen in this study as *B. xianjuensis*. The remaining portion of specimen was preserved in 70% ethanol and deposited in the Animal Specimen Museum of Wenzhou University (voucher: WZUAM20240907003; contact:Yongpu Zhang; e-mail: zhangyp@wzu.edu.cn).

### 2.2. Methods

The mitogenome of *B. xianjuensis* was sequenced using the Illumina NovaSeq 6000 platform (Novogene Bioinformatics Technology Co. Ltd., Tianjin, China) to generate paired-end 2 × 150 bp reads. The complete circular mitogenome was assembled *de novo* using NOVOPlasty 3.7 (Dierckxsens et al. 2017) and subsequently annotated using the MITOS WebServer (Bernt et al. 2013) and tRNAscan-SE (Lowe and Chan 2016). Coverage assessment following Ni et al. (2023) and Wang et al. (2023) showed an average sequencing depth of 383.028 for *B. xianjuensis* (Figure S1), indicating high assembly accuracy and completeness. The annotated mitochondrial genome has been deposited in GenBank under the accession number PX652175.

The homology search of the generated sequences was performed through nucleotide BLASTn search in the GenBank database, and based on the similarity search, the reference sequences showing highest identity matches for each of the studied species (*n* = 21) were retrieved from the GenBank. Phylogenetic analysis was performed based on the concatenated nucleotide sequences of the 13 protein-coding genes (PCGs) and two rRNA genes with a total alignment length of 17,602 bp. The analysis was conducted under the optimality criteria of Maximum Likelihood (ML) using MEGA12 (Sudhir Kumar et al. 2024) with 1000 bootstrap support and Bayesian Inference (BI) using MrBayes 3.2 (Ronquist et al. 2012). For BI, Markov Chain Monte Carlo (MCMC) was performed with four chains for 2,000,000 generations, with trees sampled every 1000 generations (the first 1000 trees were discarded as ‘burn in’). The mitochondrial genome of *Bufo gargarizans* (Cantor, 1842) (Accession number: NC_008410) was selected as the outgroup in the phylogenetic analysis.

## 3. Results and discussion

### 3.1. Characteristics of the *B. xianjuensis* mitogenome

**Figure 2.**
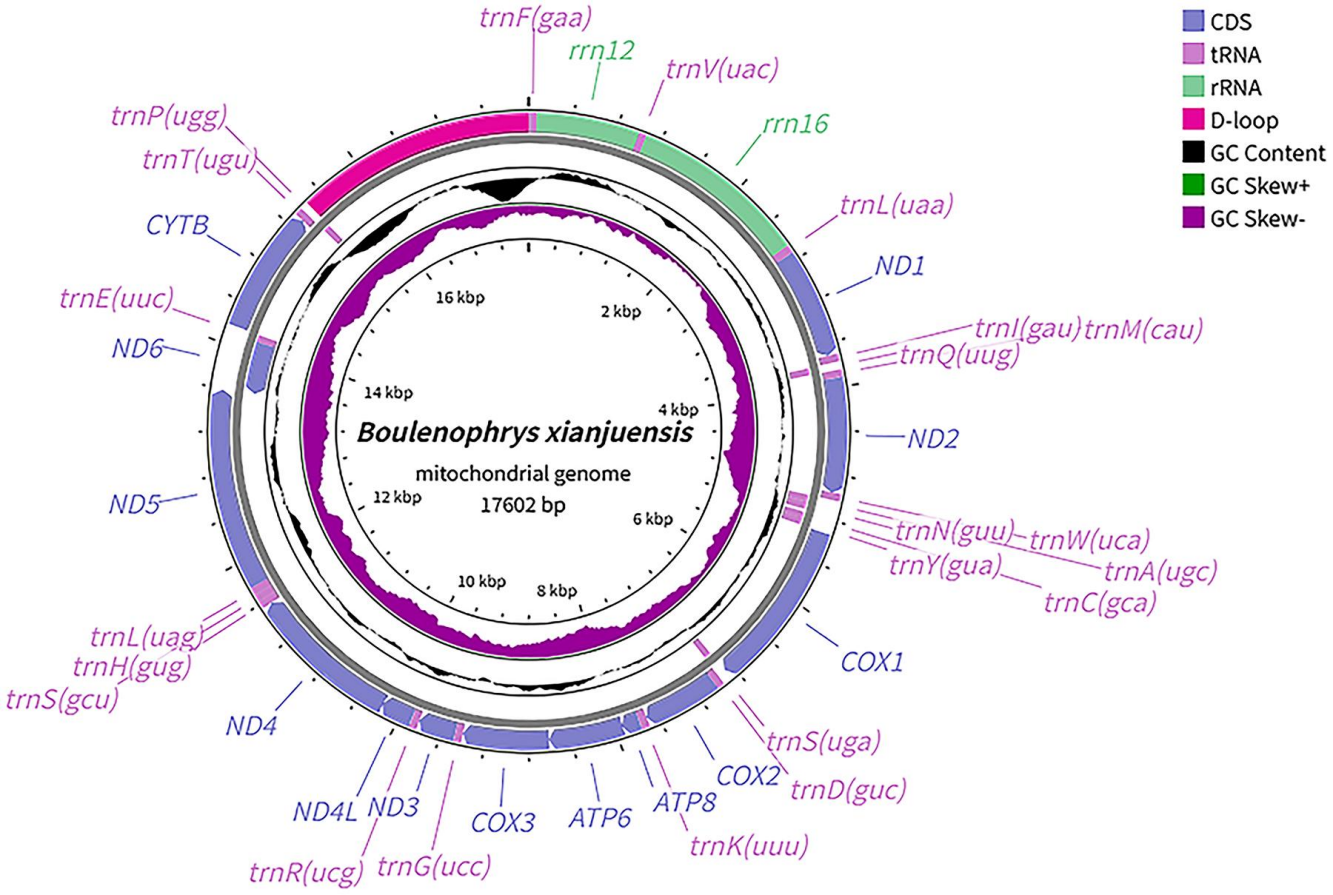
Circular map of the mitogenome of *Boulenophrys xianjuensis.*

The mitochondrial genome of *B. xianjuensis* is a circular double-stranded molecule with a length of 17,602 bp. It includes 22 tRNA genes, two rRNA genes, 13 protein-coding genes (PCGs), and a single control region (D-loop) (Figure 2). The genomic organization of the *B. xianjuensis* mitogenome is similar to that observed in other species within the genus *Boulenophrys*, such as *B. kuatunensis* (Wang et al. 2024) and *B. baishanzuensis* (Wu et al. 2024). The mitogenome has a GC content of 41.40%, with base compositions of A 27.3%, C 26.8%, G 14.7%, and T 31.2%. Most genes are encoded on the heavy strand, except for *ND6* and eight tRNA genes (*trnE*(uuc), *trnS*(tga), *trnY*(gua), *trnC*(gca), *trnN*(guu), *trnA*(ugc), *trnQ*(uug), and *trnP*(ugg)). Among the 13 PCGs, ten genes (*CYTB*, *ND6*, *ND5*, *ND4*, *ND4L*, *ATP6*, *COX3*, *ATP8*, *COX2*, and *ND1*) initiate with the canonical start codon ATG, *ND3* uses ATC, *COX1* uses GTG, and *ND2* uses ATT as their respective start codons. With regard to stop codons, three PCGs (*ND1*, *ND2*, and *ND3*) terminate with TAG, four (*COX1*, *ND4L*, *ND5*, and *ATP8*) use TAA, *ND6* terminates with AGA, and five (*COX2*, *ND4*, *CYTB*, *COX3*, and *ATP6*) are predicted to end with incomplete stop codons (a single T nucleotide).

### 3.2. Phylogenetic position

**Figure 3.**
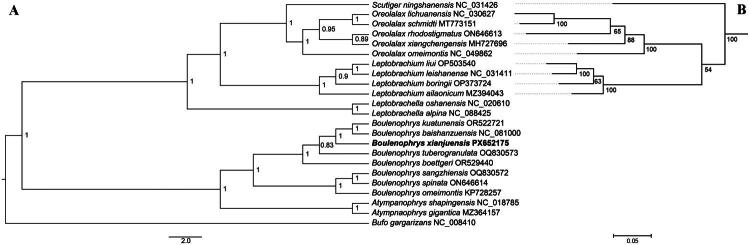
Phylogenetic hypotheses from bayesian inference (BI) and maximum likelihood (ML) analyses. Figure a shows the full phylogeny from BI, with bayesian posterior probabilities (PP ≥ 0.80) indicated at all nodes. Figure B presents a partial ML subtree highlighting only the clades with conflicting topologies compared to the BI tree, with ML bootstrap values (BS: 54-100) shown. The two trees were reconstructed based on the sequences of 13 protein-coding genes and two rRNA genes from 21 species of the family megophryidae. *Bufo gargarizans* served as the outgroup. The following sequences were used: NC_031426 (Shan et al. 2025), NC_030627 (Wang et al. 2016), MT773151 (Bao et al. 2020), ON646613 (unpublished), MH727696 (Li et al. 2018), NC_049862 (unpublished), OP503540 (Zhou et al. 2023), NC_031411 (Liang et al. 2016), OP373724 (Zhou et al. 2023), MZ394043 (unpublished), NC_020610 (Xiang et al. 2013), NC_088425 (Shu et al. 2021), OR522721 (Wang et al. 2024), NC_081000 (Wu et al. 2024), OQ830573 (Xiang et al. 2024), OR529440 (Wang et al. 2024), OQ830572 (Xiang et al. 2024), ON646614 (unpublished), KP728257 (Liu et al. 2016), NC_018785 (Xiang et al. 2013), MZ364157 (unpublished), and NC_008410 (Jiang et al. 2017). The samples sequenced in this study are highlighted in bold.

Phylogenetic reconstructions based on both BI and ML methods yielded largely congruent topologies. The BI tree was fully resolved and is presented as the primary phylogenetic hypothesis. Notably, the focal genera *Boulenophrys* and *Atympanophrys* formed a highly supported, monophyletic clade in both analyses, with all internal nodes receiving strong support (PP = 0.83-1.00, BS = 80-100) (Figure 3). This provides robust confirmation of the placement of *B. xianjuensis* within *Boulenophrys* and supports the sister-group relationship between *Boulenophrys* and *Atympanophrys*. Although the monophyly of the genera *Oreolalax*, *Scutiger*, *Leptobrachium*, and *Leptobrachella* was consistently recovered, the intergeneric branching order among these taxa differed between the BI and ML topologies, reflecting uncertainty in their deep phylogenetic relationships. Despite this limited topological discordance, the core findings concerning the systematics of *Boulenophrys* and *Atympanophrys* are strongly and consistently supported.

## 4. Discussion and conclusion

DNA barcoding technology serves as an accurate and effective tool for species identification, particularly in cases where morphological characteristics are insufficient for precise taxonomic assignment during field surveys. In this study, we demonstrate that the gene arrangement and genomic composition of the *B. xianjuensis* mitochondrial genome are highly conserved and consistent with those observed in other species within the genus *Boulenophrys* (Wu et al. 2024). The taxonomic status of *Boulenophrys* has historically been debated, with some studies treating it as a subgenus of *Megophrys* (Mahony et al. 2017), while others recognize it as a distinct genus (Chen et al. 2017). This taxonomic ambiguity was recently resolved by Lyu et al. (2023), who proposed a revised classification of the subfamily Megophryinae, elevating it to include 10 genera—among them *Boulenophrys*—based on comprehensive phylogenetic and morphological evidence. Expanding the availability of mitochondrial genomes across Megophryinae is essential for validating and refining this updated taxonomic framework. The present study reports the first complete mitochondrial genome of *B. xianjuensis*, providing critical molecular data for addressing phylogenetic relationships and genetic evolutionary questions within this species and its ader taxonomic group.

## Supplementary Material

coverage_plot.tif

ARRIVE Checklist.pdf

Supplementary material.docx

## Data Availability

The genome sequence data that support the findings of this study are openly available in GenBank of NCBI at https://www.ncbi.nlm.nih.gov/ under the accession no. PX652175.

## References

[CIT0001] Bao X et al. 2020. The near complete mitochondrial genome of *Oreolalax schmidti* (Anura: megophryidae). Mitochondrial DNA B Resour. 5(3):3536–3537. 10.1080/23802359.2020.1806134 33458231 PMC7782216

[CIT0002] Bernt M et al. 2013. MITOS: improved *de novo* metazoan mitochondrial genome annotation. Mol Phylogenet Evol. 69(2):313–319. 10.1016/j.ympev.2012.08.023 22982435

[CIT0003] Chen JM et al. 2017. A novel multilocus phylogenetic estimation reveals unrecognized diversity in Asian horned toads, genus *Megophrys sensu lato* (Anura: megophryidae). Mol Phylogenet Evol. 106:28–43. 10.1016/j.ympev.2016.09.004 27622725

[CIT0004] Dierckxsens N, Mardulyn P, Smits G. 2017. NOVOPlasty: *de novo* assembly of organelle genomes from whole genome data. Nucleic Acids Res. 45(4):e18. 10.1093/nar/gkw955 28204566 PMC5389512

[CIT0005] Frost DR. 2025. Amphibian species of the world: an online reference, version 6.0. New York (NY): American Museum of Natural History; [accessed date 2025 Oct 15]. http://research.amnh.org/herpetology/amphibia/index.php/

[CIT0006] Jiang L, Liu Y, Zhao L, Ruan Q. 2017. Complete mitochondrial genome sequence of the Asiatic Toad *Bufo Gargarizans* (Amphibia, Anura, Bufonidae). Mitochondrial DNA B Resour. 2(2):836–838. 10.1080/23802359.2017.1407700 33474003 PMC7800167

[CIT0007] Kumar S et al. 2024. MEGA12: molecular evolutionary genetic analysis version 12 for adaptive and green computing. Mol Biol Evol. 41(12):1–9. 10.1093/molbev/msae263 PMC11683415 39708372

[CIT0008] Li S, Gao X, Wei G, Wang B, Xu N. 2018. The complete mitochondrial genome of the toad species *Oreolalax xiangchengensis* (Anura: megophryidae) and phylogenetic analyses. Mitochondrial DNA B Resour. 3(2):1298–1299. 10.1080/23802359.2018.1535842 33474500 PMC7799451

[CIT0009] Liang XX et al. 2016. Complete mitochondrial genome of the Leishan moustache toad, *Vibrissaphora leishanensis* (Anura: megophryidae). Mitochondrial DNA B Resour. 1(1):275–276. 10.1080/23802359.2016.1159937 33644358 PMC7871820

[CIT0010] Liu J et al. 2016. The near-complete mitogenome sequence of the Omei Horned Toad *Megophrys omeimontis* Liu, 1950 (Anura, Megophryidae). Mitochondrial DNA A DNA Mapp Seq Anal. 27(4):2389–2390. 10.3109/19401736.2015.1028044 25856516

[CIT0011] Lowe TM, Chan PP. 2016. tRNAscan-SE On-line: integrating search and context for analysis of transfer RNA genes. Nucleic Acids Res. 44(W1):W54–W57. 10.1093/nar/gkw413 27174935 PMC4987944

[CIT0012] Lyu ZT et al. 2023. Generic classification of Asian horned toads (Anura: megophryidae: megophryinae) and monograph of Chinese species. Zool Res. 44(2):380–450. 10.24272/j.issn.2095-8137.2022.372 36924402 PMC10083232

[CIT0013] Mahony S, Foley NM, Biju SD, Teeling EC. 2017. Evolutionary history of the Asian horned frogs (Megophryinae): integrative approaches to timetree dating in the absence of a fossil record. Mol Biol Evol. 34(3):744–771. 10.1093/molbev/msw267 28100792

[CIT0014] Ni Y, Li J, Zhang C, Liu C. 2023. Generating sequencing depth and coverage map for organelle genomes. protocols.io. 10.17504/protocols.io.4r3l27jkxg1y/v1

[CIT0015] Ronquist F et al. 2012. MrBayes 3.2: efficient Bayesian phylogenetic inference and model choice across a largemodel space. Softw Syst Evol. 61(3):539–542. 10.1093/sysbio/sys029 PMC3329765 22357727

[CIT0016] Shan S et al. 2025. Mitochondrial genome of *Scutiger ningshanensis* (Anura, Megophryidae, Scutiger): insights into the characteristics of the mitogenome and the phylogenetic relationships of Megophryidae species. Genes (Basel). 16(8):879. 10.3390/genes16080879 40869927 PMC12385210

[CIT0017] Shu G, Yu M, He Z, Xie F, Liang X. 2021. Complete mitochondrial genome of the Alpine Metacarpal-tubercled Toad *Leptobrachella alpina* (Amphibia, Anura, Megophryidae). Mitochondrial DNA B Resour. 6(11):3242–3243. 10.1080/23802359.2021.1990149 34693010 PMC8530483

[CIT0018] Wang B et al. 2020. A new *Megophrys* Kuhl & Van Hasselt (Amphibia, Megophryidae) from southeastern China. Zookeys. 904:35–62. 10.3897/zookeys.904.47354 31997889 PMC6978424

[CIT0019] Wang G et al. 2016. The complete mitochondrial genome of *Oreolalax lichuanensis* (Amphibia, Anura, Megophryidae). Mitochondrial DNA B Resour. 1(1):905–906. 10.1080/23802359.2016.1143340 33473673 PMC7800913

[CIT0020] Wang JY et al. 2023. How does mitochondrial protein-coding gene expression in *Fejervarya kawamurai* (Anura: dicroglossidae) respond to extreme temperatures?. Animals (Basel). 13(19):3015. 10.3390/ani13193015 37835622 PMC10571990

[CIT0021] Wang ZY et al. 2024. Two complete mitochondrial genomes of *Boulenophrys* (Anura: megophryidae: megophryinae): characteristics and phylogenetic implications. Mitochondrial DNA B Resour. 9(8):1098–1102. 10.1080/23802359.2024.2392745 39165385 PMC11334740

[CIT0022] Wu JP et al. 2024. The complete mitochondrial genome of the Baishanzu horned toad *Boulenophrys baishanzuensis* (Anura: megophryidae). Mitochondrial DNA B Resour. 9(1):209–213. 10.1080/23802359.2024.2307995 38298222 PMC10829807

[CIT0023] Xiang H et al. 2024. Insights into phylogenetic positions and distribution patterns: complete mitogenomes of two sympatric Asian horned toads in *Boulenophrys* (Anura: megophryidae). Ecol Evol. 14(7):e11687. 10.1002/ece3.11687 38994208 PMC11237341

[CIT0024] Xiang T, Wang B, Jiang J, Li C, Xie F. 2013. The complete mitochondrial genome of *Megophrys shapingensis* (Amphibia, Anura, Megophryidae). Mitochondrial DNA. 24(1):43–45. 10.3109/19401736.2012.717936 22954176

[CIT0025] Xiang TM et al. 2013. Complete mitochondrial genome of *Paramegophrys oshanensis* (Amphibia, Anura, Megophryidae). Mitochondrial DNA. 24(5):472–474. 10.3109/19401736.2013.766183 23391261

[CIT0026] Zhou Q et al. 2023. Two complete mitochondrial genomes of *Leptobrachium* (Anura: megophryidae: leptobrachiinae): characteristics, population divergences, and phylogenetic implications. Genes (Basel). 14(3):768. 10.3390/genes14030768 36981038 PMC10048368

