# Supplementary material for: The complete mitochondrial genome of the Boulenophrys xianjuensis (Anura: Megophryidae)
Source: Mitochondrial DNA B Resour. 2026 Jun 25;11(7):879–82. doi: 10.1080/23802359.2026.2694182 (PMC13307374; doi:10.1080/23802359.2026.2694182)
Supplement: Supplementary material.docx [file TMDN_A_2694182_SM6067.docx]

**Supplementary material**

**The complete mitochondrial genome of the *Boulenophrys xianjuensis* (Anura: Megophryidae)**

Yingying Xing, Huimin Pei, Haili Ou, Lie Xie, YongPu Zhang





**Figure S1.** Coverage maps of the mitochondrial genomes for *Boulenophrys xianjuensis*
